# Identification of *cis-* and *trans*-acting elements regulating calretinin expression in mesothelioma cells

**DOI:** 10.18632/oncotarget.7114

**Published:** 2016-02-01

**Authors:** Jelena Kresoja-Rakic, Esra Kapaklikaya, Gabriela Ziltener, Damian Dalcher, Raffaella Santoro, Brock C. Christensen, Kevin C. Johnson, Beat Schwaller, Walter Weder, Rolf A. Stahel, Emanuela Felley-Bosco

**Affiliations:** ^1^ Laboratory of Molecular Oncology, Clinic of Oncology, University Hospital Zürich, Zürich, Switzerland; ^2^ Institute of Veterinary Biochemistry and Molecular Biology, University of Zürich, Zürich, Switzerland; ^3^ Departments of Epidemiology, Pharmacology and Toxicology and Community and Family Medicine, Geisel School of Medicine at Dartmouth, Hanover, NH, USA; ^4^ Anatomy, Department of Medicine, University of Fribourg, Fribourg, Switzerland; ^5^ Division of Thoracic Surgery, University Hospital Zürich, Zürich, Switzerland

**Keywords:** malignant pleural mesothelioma, calretinin, promoter, NRF-1, cell-cycle regulated expression

## Abstract

Calretinin (*CALB2*) is a diagnostic marker for epithelioid mesothelioma. It is also a prognostic marker since patients with tumors expressing high calretinin levels have better overall survival. Silencing of calretinin decreases viability of epithelioid mesothelioma cells. Our aim was to elucidate mechanisms regulating calretinin expression in mesothelioma. Analysis of calretinin transcript and protein suggested a control at the mRNA level. Treatment with 5-aza-2′-deoxycytidine and analysis of TCGA data indicated that promoter methylation is not likely to be involved. Therefore, we investigated *CALB2* promoter by analyzing ~1kb of genomic sequence surrounding the transcription start site (TSS) + 1 using promoter reporter assay. Deletion analysis of *CALB2* proximal promoter showed that sequence spanning the −161/+80bp region sustained transcriptional activity. Site-directed analysis identified important *cis*-regulatory elements within this −161/+80bp *CALB2* promoter. EMSA and ChIP assays confirmed binding of NRF-1 and E2F2 to the *CALB2* promoter and siRNA knockdown of NRF-1 led to decreased expression of calretinin. Cell synchronization experiment showed that calretinin expression was cell cycle regulated with a peak of expression at G1/S phase. This study provides the first insight in the regulation of *CALB2* expression in mesothelioma cells.

## INTRODUCTION

Malignant pleural mesothelioma (MPM) are tumors originating from the surface serosal cells of the pleura [1]. With the established standard of care, i.e. pemetrexed with platinum-based chemotherapy, MPM patients have a median survival of one year [2], therefore it is important to better understand the biology of mesothelioma development that would allow for designing and developing novel and more effective therapy strategies.

Calretinin (CR, gene *CALB2*) is a ~30kDa calcium-binding protein belonging to the EF-hand family [3]. In 1996, Doglioni et al. demonstrated specificity of calretinin immunoreactivity in mesothelioma compared to other tumor types [4]. Currently, calretinin is the most commonly used diagnostic mesothelioma marker [5]. Reactive mesothelial cells and malignant mesothelioma cells show nuclear and cytosolic calretinin staining allowing differentiation of mesothelioma from adenocarcinoma [6]. MPM can be categorized into three groups based on the histological characteristics: epithelial, biphasic and sarcomatoid characterized by strong, intermediate and weak (absent) calretinin immunoreactivity, respectively [7]. Three independent studies suggested that calretinin can be also utilized as a prognostic marker since strong calretinin immunostaining correlated with improved survival [7–9].

In two epithelioid mesothelioma cell lines, down-regulation of calretinin by a lentiviral short hairpin approach impaired cell proliferation and triggered apoptosis via the intrinsic caspase-9-dependent pathway [10] suggesting that calretinin is important for epithelioid mesothelioma cell survival.

Calretinin is physiologically expressed in different neuronal cell populations, e.g. in the retina and the cerebellum [11] as well as in Leydig cells, Sertoli cells and adipocytes [5]. Little is known about the mechanisms regulating calretinin expression in various tissues or in cancer. It has been reported that the mouse *Calb2* and human *CALB2* promoter region contain TATA and CAAT boxes [12]. A mouse *Calb2* promoter fragment (−115/*+*54bp) was shown to be active in neuronal and cancer cells [13, 14]. In human colon cancer cells, calretinin expression is downregulated by butyrate, a substance that induces cell differentiation [15]. Butyrate–induced calretinin downregulation is mediated through butyrate dependent repressive elements which are not operational in mesothelioma cells [16].

In this study, our aim was to investigate and characterize transcriptional control of calretinin expression in mesothelioma cells.

## RESULTS

### Calretinin protein levels correlate with *CALB2* transcript levels across a panel of different mesothelioma cell lines

**Figure 1:**
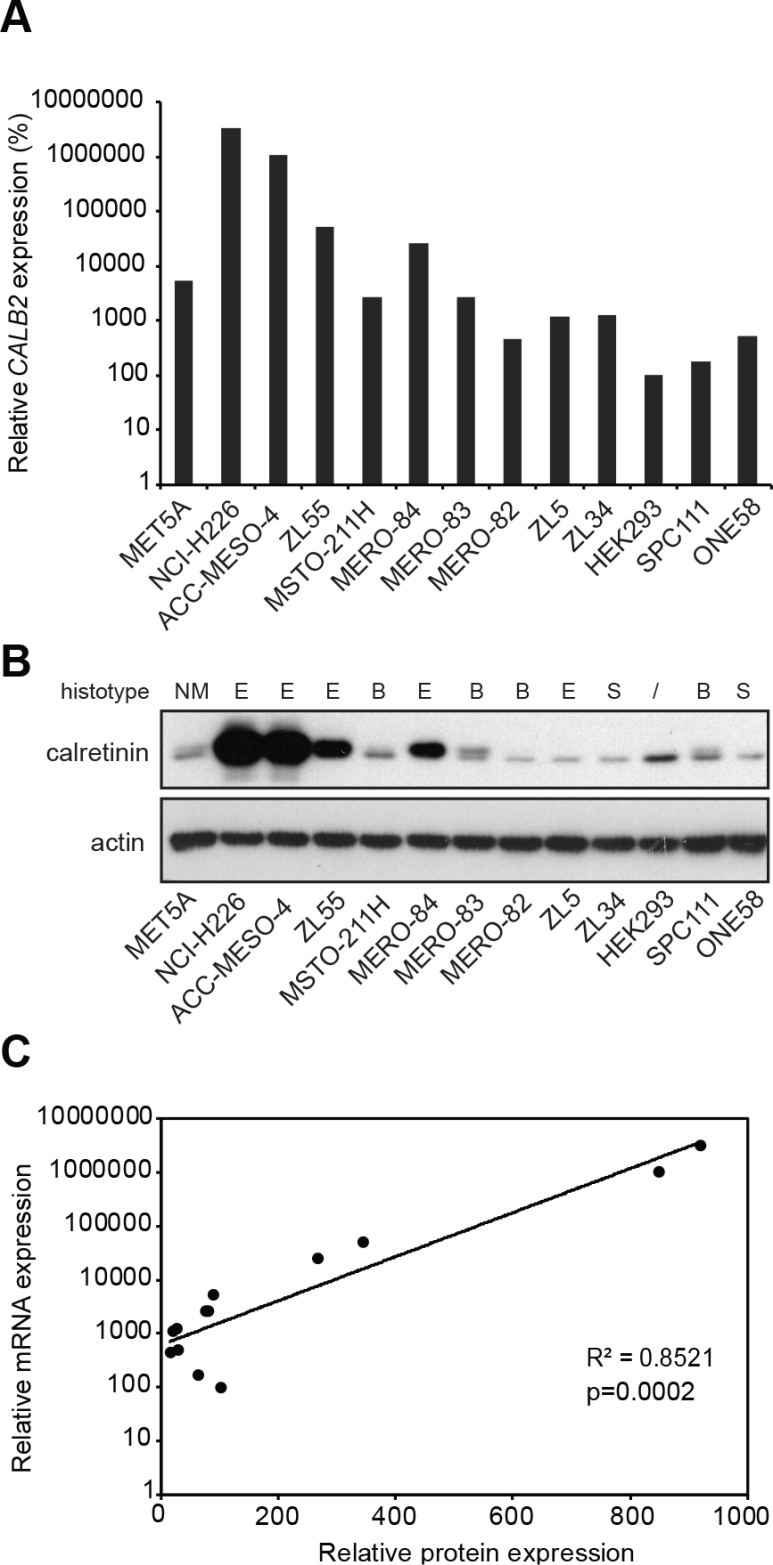
Differential expression of calretinin in a panel of 13 cell lines (**A**) Quantitative RT-PCR analysis of *CALB2* expression in 11 mesothelioma cell lines, one immortalized mesothelial cell line (MET5A) and HEK293 cells using histones as an internal control. Levels are shown relative to the HEK293 cells according to the –ΔΔCt method. (**B**) Western blot analysis of calretinin protein levels in the same panel of cell lines. Actin was used as loading control. (**C**) Relative mRNA levels are plotted against the relative protein levels; each dot represents a cell lines as in A and B.

To characterize calretinin expression, we assessed mRNA and protein levels across a panel consisting of 11 mesothelioma cell lines, one SV-40 immortalized human pleural mesothelial cell line (MeT5A) and HEK293 cells (Figure 1A and 1B). Five cell lines were of epithelioid type (NCI-H226, ACC-MESO-4, ZL55, MERO-84, and ZL5), four were biphasic (MSTO-211H, MERO-82, MERO-83, SPC111) and two were sarcomatoid (ZL34 and ONE58). Levels of *CALB2* transcript were significantly higher (*p =* 0.0285) in epithelioid histotype. Calretinin was also expressed in HEK293 cells, which might be expected since HEK293 cells are of kidney embryonic origin [17] and both kidney and mesothelium originate from the mesoderm. Importantly, *CALB2* mRNA expression was strongly positively (*p =* 0.0002) correlated with calretinin protein levels (Figure 1C), suggesting that calretinin expression could be regulated either through copy number variation or through control of mRNA levels.

### Calretinin promoter is not inhibited by DNA methylation in mesothelioma cell lines and tumor samples

Analysis of genomic copy number abnormalities (CNA) in mesothelioma, using arrayMap [18] showed no indications of genetic alteration in *CALB2* gene (Supplementary Figure 1) while a study has described loss at 16q22 in two out of 18 mesothelioma cases [19], indicating that upregulation of calretinin expression in mesothelioma is not linked to increased gene copy number.

**Figure 2:**
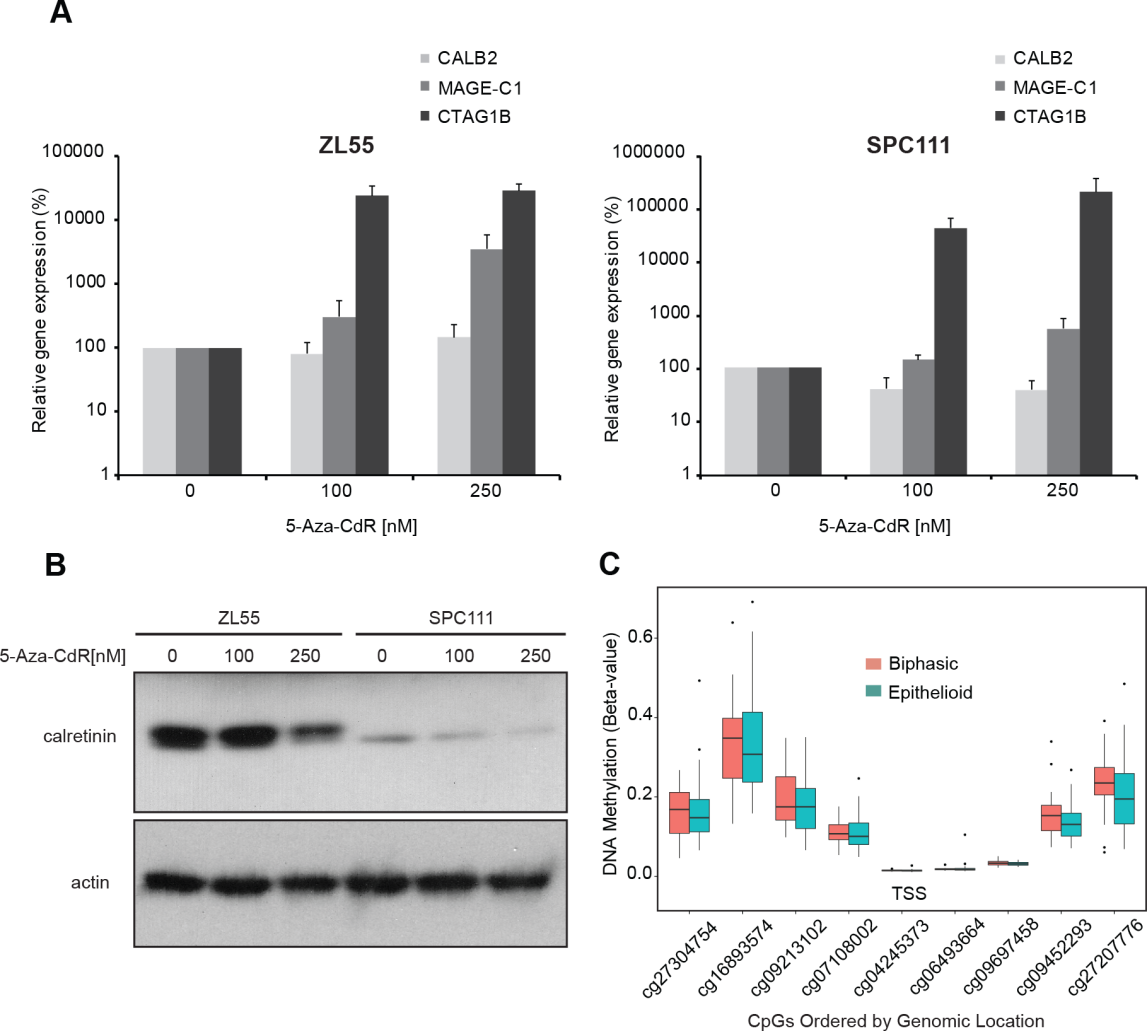
Promoter methylation does not regulate calretinin expression in cell lines and tumor samples (**A**) ZL55 and SPC111 cells were treated for 7 days with the hypomethylating agent 5-Aza-CdR (100 nM and 250 nM). Analysis of mRNA showed no change in *CALB2* expression but strong upregulation of *MAGE-C1* and *CTAG1B* expression. Mean ± SD, *n =* 3. (**B**) Calretinin protein levels decreased upon 5-Aza-CdR treatment in both ZL55 and SPC111 cells. Representative of three independent experiments. (**C**) CpG sites in the promoter region of *CALB2* from the Illumina HumanMethylation450 array are shown in 5′ to 3′ order versus methylation beta-value which represents the percent methylation of the sample. Mesotheliomas are stratified by tumor histology, epithelioid (*n =* 57), and biphasic (*n =* 23).

We then took advantage of the known differential expression of calretinin between epithelioid and sarcomatoid mesothelioma to explore whether this might represent a hint that calretinin expression is controlled by methylation of the promoter, since this mechanism controls the expression of several genes in MPM [20]. A putative *CALB2* proximal promoter region was defined based on two criteria: the observation that most human promoters are found between −800 upstream and *+*200 bp downstream of putative transcription start site (TSS; *+*1) [21] and according to publicly available data on high throughput ChIP data reported in the UCSC browser (Supplementary Figure 2). *In silico* analysis (http://www.bioinformatics.org/sms2/cpg_islands.html) of the −838/*+*80bp region of *CALB2* promoter using the method defined by Gardiner-Garden [22], documented the presence of CpG islands and a high GC content starting from 338bp upstream of the TSS. Inactivation of gene expression by methylation of CpG islands present in promoters is a common epigenetic mechanism in health and diseases [23]. Therefore, to test the hypothesis that calretinin expression might be partly driven by epigenetic mechanisms, ZL55 (high-calretinin, epithelioid) and SPC111 (low-calretinin, biphasic) cells were treated for seven days with the hypomethylating agent 5-aza-2′-deoxycytidine (5-Aza-CdR) at 100 and 250 nM and the expression of calretinin was evaluated. The expression of two cancer-associated testis antigens *CTAG1B* and *MAGE-C1* genes was used as a positive control, since their promoters are known to be controlled by DNA methylation [24]. Although the expression of *CTAG1B* and *MAGE-C1* mRNA was strongly enhanced by 5-Aza-CdR treatment in SPC111 and ZL55 cells (Figure 2A), the expression of calretinin mRNA and protein (Figure 2A and 2B) did not increase. On the contrary, treatment with 5-Aza-CdR resulted in a decrease in calretinin protein levels, especially in SPC111 cells. Moreover, the methylation status of nine CpG sites in the *CALB2* promoter of epithelioid (*n =* 57) and biphasic (*n =* 23) mesothelioma samples from The Cancer Genome Atlas (TCGA) database generally showed low methylation levels, particularly at CpG sites nearest to the TSS (Figure 2C). *CALB2* promoter CpG methylation was not significantly negatively correlated with *CALB2* gene expression in epithelioid or biphasic tumors (Table 1). As control, the methylation status of *MAGEC1* promoter was also investigated. The region of the *MAGEC1* promoter exhibits high levels of methylation (Supplementary Figure 3) and the gene is lowly expressed in TCGA mesothelioma tumors. Importantly, several of the CpGs located at or near the transcriptional start site are significantly negatively correlated with gene expression (Supplementary Table 2) and the gene is lowly expressed in both biphasic and epithelioid tumors. The high levels of DNA methylation and low levels of expression coupled with our experimental evidence of reactivation of gene expression with treatment with hypomethylating agents suggest that *MAGEC1* serves as an appropriate control gene in our study. Taken together, our data suggest that promoter methylation is not driving differential expression of calretinin between mesothelioma histotypes.

**Table 1:** Correlation between CALB2 gene promoter CpG methylation and CALB2 expression (*Spearman's test)

cgID	Chromosome	Position	Epithelioid Correlation	Epithelioid *P*-value*	Biphasic Correlation	Biphasic *P*-value*
cg27304754	16	71392088	0.12	0.385	0.45	0.034
cg16893574	16	71392095	−0.14	0.296	0.42	0.047
cg09213102	16	71392373	−0.14	0.312	0.35	0.122
cg07108002	16	71392377	−0.24	0.068	0.23	0.285
cg04245373	16	71392615	0.02	0.893	−0.26	0.223
cg06493664	16	71392628	0.04	0.749	−0.41	0.053
cg09697458	16	71392748	−0.09	0.502	0.05	0.823
cg09452293	16	71392838	−0.16	0.230	0.05	0.823
cg27207776	16	71393801	−0.25	0.063	−0.07	0.740

### The −161/+80bp *CALB2* promoter region drives expression of luciferase reporter in mesothelioma cells

**Figure 3:**
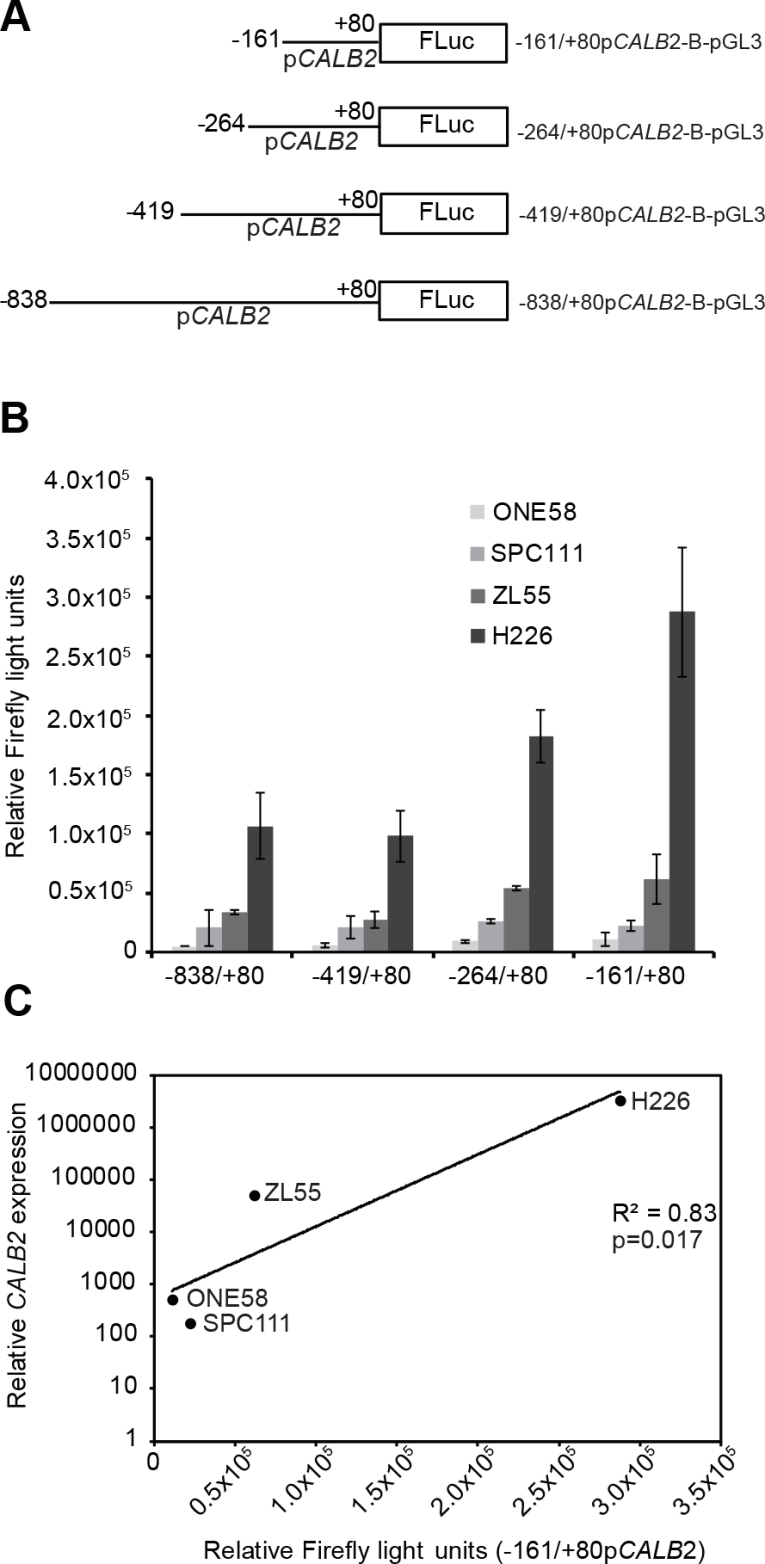
Transcriptional activity of the minimal *CALB2* promoter is proportional to calretinin expression Transcriptional activity of different promoter constructs was assessed by transient transfection and dual luciferase assay using pTK-RL as an internal control. (**A**) 5′-deletion constructs of the *CALB2* promoter engineered in the pGL3-basic (pGL3-B) luciferase reporter plasmid. Lines represent *CALB2* promoter fragment; the name and the size are indicated by nucleotide position upstream (−) or downstream (*+*) relative to the transcription start site (*+*1). (**B**) *CALB2* promoter activity in ONE58, SPC111, ZL55 and NCI-H226 cells, which express different levels of calretinin. To allow for comparison of the absolute promoter activity between the cell lines, Firefly light units were multiplied by a correction factor that compensates for differences in the transfection efficacy. The correction factor was based upon the Renilla Light Units (ReLU) of the internal control, pTK-RL, and was calculated by dividing the mean ONE58 ReLU by the mean of ZL55, SPC111 and H226 ReLU for each construct. Mean ± SD, *n =* 3. (**C**) Relative mRNA abundance is plotted versus the relative Firefly light units reflecting absolute −161/*+*80bp *CALB2* activity for the indicated cell lines.

We then investigated the activity of the *CALB2* promoter to define *cis*-regulatory sequences important for calretinin expression. Four 5′-deletion promoter fragments (−838, −419, −264, −161bp upstream and *+*80bp downstream of TSS *+*1) were cloned into the firefly luciferase basic reporter pGL3-B (Figure 3A). To test transcriptional activity of the promoter constructs, NCI-H226, Zl55, SPC111 and ONE58 cells were transiently transfected with the promoter constructs along with the pRL-TK plasmid as an internal control. The shortest −161/*+*80bp *CALB2* promoter construct appeared to be as active as the other longer sequences in all tested cell lines (Figure 3B) defining this fragment as the minimal *CALB2* promoter. The 240bp minimal promoter has more activity than any of the longer versions (Supplementary Figure 4) indicating the presence of negative regulators. In addition, −161/*+*80bp *CALB2* promoter activity was correlated (*p =* 0.017) with *CALB2* expression (Figure 3C) with the lowest transcriptional activity observed in ONE58 cells which express extremely low levels of calretinin, and the highest transcriptional activity in NCI-H226 cells, expressing the highest levels of calretinin. Since the −161/*+*80bp fragment was sufficient to drive reporter expression in the tested mesothelioma cells, the next step was to further characterize its *cis*-regulatory elements.

### Mutation of E2F2/NRF-1-like site strongly reduced promoter activity

**Figure 4:**
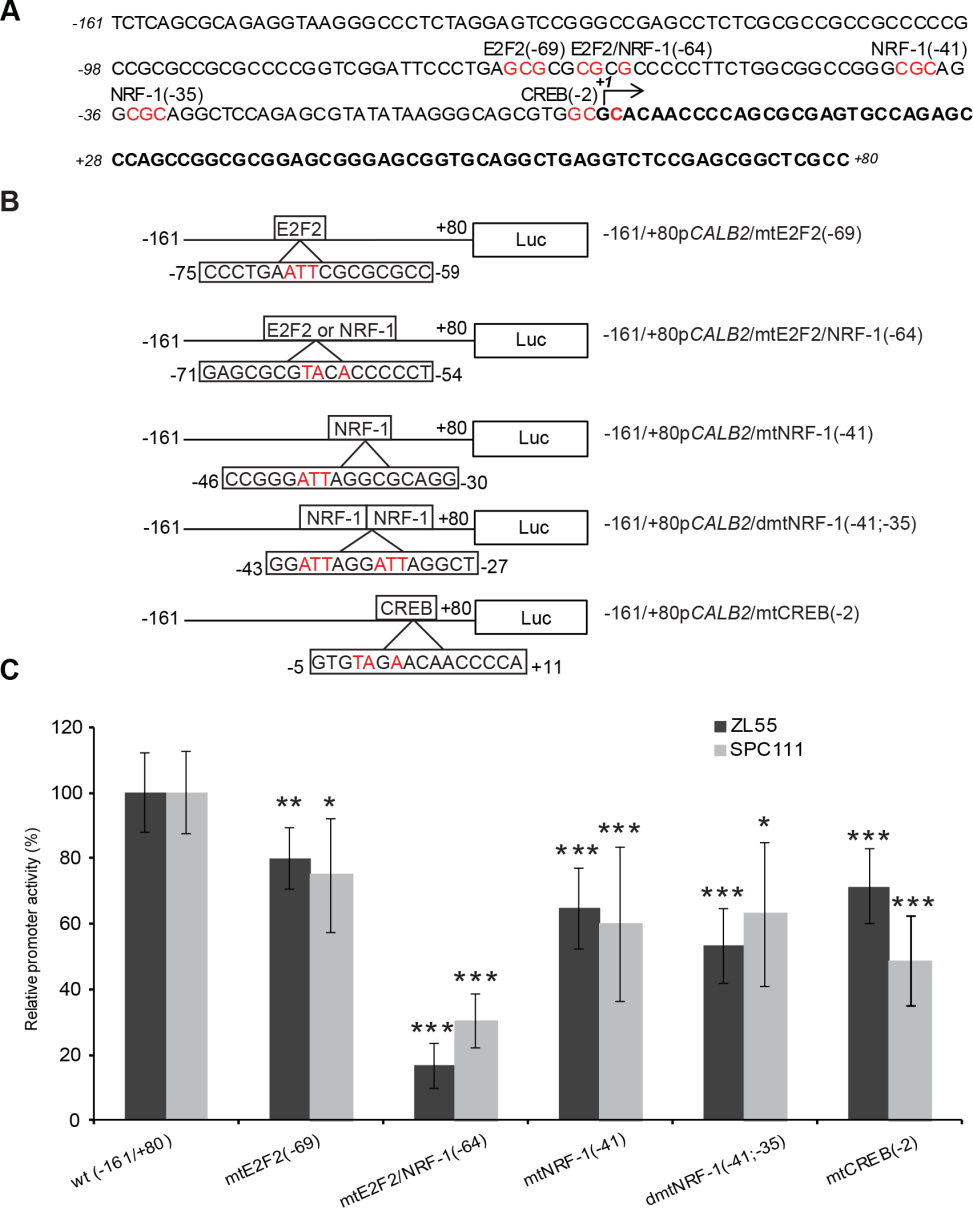
Effect of site-directed mutagenesis of transcription factor binding sites on the −161/+80bp *CALB2* promoter activity (**A**) Predicted potential TF binding sites (highly conserved nucleotides selected for site-directed mutagenesis are colored in red). (**B**) A series of mutant constructs harboring mutated nucleotides (in red) of predicted E2F2, NRF-1 and CREB binding sites. Numbers indicate the position of the first (from the left to the right) mutated nucleotide. (**C**) The wild-type reporter plasmid expression was arbitrarily set to 100% and the reporter activity of the mutants was expressed as a percentage of the wild-type construct. Mean ± SD *n =* 3; *p < 0.05; **p < 0.01; ***p < 0.005.

To search for putative protein binding sites, *in silico* analysis was performed on the sequence from −161bp to *+*80bp of the *CALB2* promoter using MatInspector software [25]. Most frequently identified binding sites were the ones for NRF-1 (nuclear respiratory factor 1), as well as for E2F family members and CREB (cAMP response element binding protein) (Figure 4A). The activation of the latter has been observed *in vitro* after exposure of mesothelial cells to asbestos [26]. Promoter constructs containing selective mutations were created using a site-directed mutagenesis approach (Figure 4B) and their transcriptional activity was measured in ZL55 and SPC111 cells. All mutated promoter constructs showed significantly decreased transcriptional activity compared to the control construct in both cell lines (Figure 4C). The transcriptional activity of the E2F2/NRF-1 (−64)-like mutant resulted in a 80% decrease compared to the wild-type promoter sequence suggesting that E2F2 or NRF-1 might be important for the transcriptional control of calretinin expression. A strongly reduced activity of the E2F2/NRF-1 mutant construct was also observed in HEK293 cells (Supplementary Figure 5), indicating that this transcriptional control is not restricted to mesothelioma cells, but possibly to other cells of mesodermal origin.

### *In vitro* and *in situ* binding of NRF-1 and E2F2 to E2F2/NRF-1-like binding motif in the *CALB2* promoter

**Figure 5:**
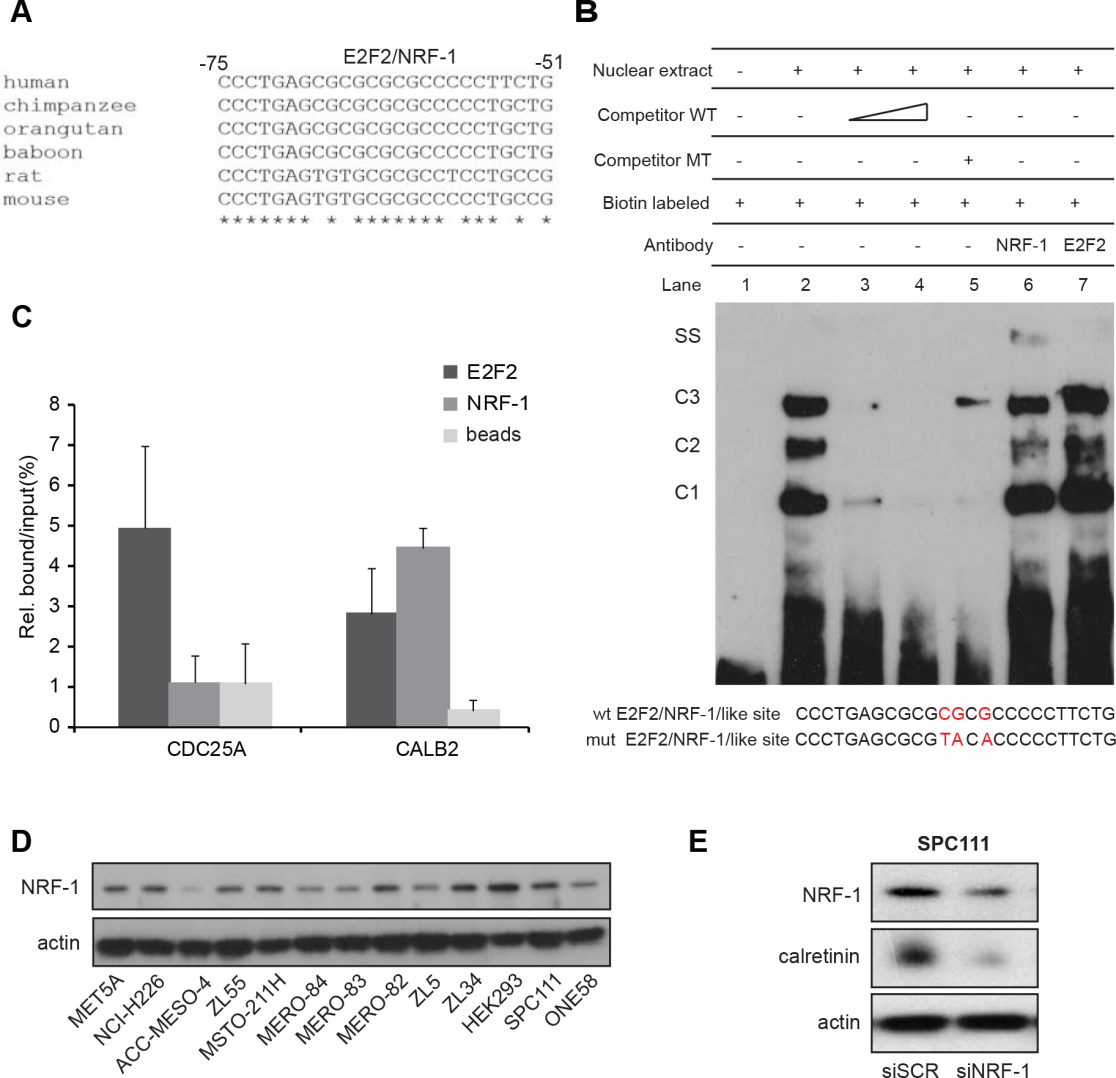
NRF-1 and E2F2 bind to the *CALB2* promoter (**A**) Nucleotide sequence comparison reveals high homology in the *CALB2* promoter regions of different species in the stretch containing E2F2/NRF-1 predicted binding sites. The analysis was performed using the ClustalW2 software. Asterisks indicate conserved nucleotides. (**B**) EMSA assay showing that a 25bp-oligo containing E2F2/NRF-1-like sites forms three DNA-protein complexes (C1, C2, C3) when incubated in with the ZL55 cells-derived nuclear extracts. Addition of a NRF-1 antibody but not of one against E2F2 resulted in a supershift (SS). (**C**) qPCR analysis on *CDC25A* and *CALB2* promoter regions after ChIP-experiments. Data of two independent experiments were normalized to input. (**D**) NRF-1 protein expression in different cell lines. Actin is used as loading control. (**E**) NRF-1 silencing resulted in downregulation of calretinin expression.

Alignment of the *CALB2* promoter ortholog nucleotide sequences (Supplementary Figure 6) revealed that the stretch containing the putative binding sites for E2F2 or NRF-1 (−75 to −51bp) shows the highest degree of identity across various species (Figure 5A). Therefore, to experimentally demonstrate potential functionally relevant binding of E2F2 or NRF-1, an EMSA assay was performed using biotin labeled 25-bp oligonucleotide containing the putative E2F2/NRF-1 binding site (Figure 5B). Upon incubation of ZL55 nuclear extract with the specific labeled probe, DNA-protein complexes C1, C2 and C3 were formed (Figure 5B, lane 2). All three DNA-protein complexes were diminished with increasing amounts of unlabeled competitor (Figure 5B, lane 3 and 4) but were not outcompeted using an unrelated oligo competitor (data not shown). However, in the reaction with mutated competitor, the C3 complex was detected again but not the complexes C1 and C2 (Figure 5B, lane 5). Addition of a specific antibody against NRF-1 resulted in the appearance of a heavier complex (Figure 5B, lane 6) and a decrease of the C2 complex, while no change was observed with an anti-E2F2 antibody (Figure 5B, lane 7).

To confirm *in situ* in cultured cells the presence of NRF-1 or E2F2 on the *CALB2* promoter, chromatin immunoprecipitation assay (ChIP) was performed in ZL55 cells with antibodies against NRF-1 and E2F2. The promoter of *CDC25A* gene, a known E2F target [27] lacking a binding site for NRF-1, was used as a negative control for NRF-1 and as a positive control for E2F2. Chromatin immunoprecipitation assay showed that compared to *CDC25A*, NRF-1 was significantly enriched in *CALB2* promoter whereas E2F2 bound to both, *CDC25A* and *CALB2* promoters (Figure 5C). These data demonstrate that NRF-1 and E2F2 are able to bind to *CALB2* promoter.

NRF-1 expression levels are likely not responsible for differences in calretinin expression levels observed in the various MPM lines since NRF-1 levels were rather homogenous in the tested cell lines (Figure 5D). However, silencing of NRF-1 resulted in calretinin downregulation in SPC111 cells (Figure 5E and Supplementary Figure 7). Taken together, these data provide evidence that NRF-1 is an important positive regulator for calretinin expression.

### Calretinin expression is cell cycle-dependent

**Figure 6:**
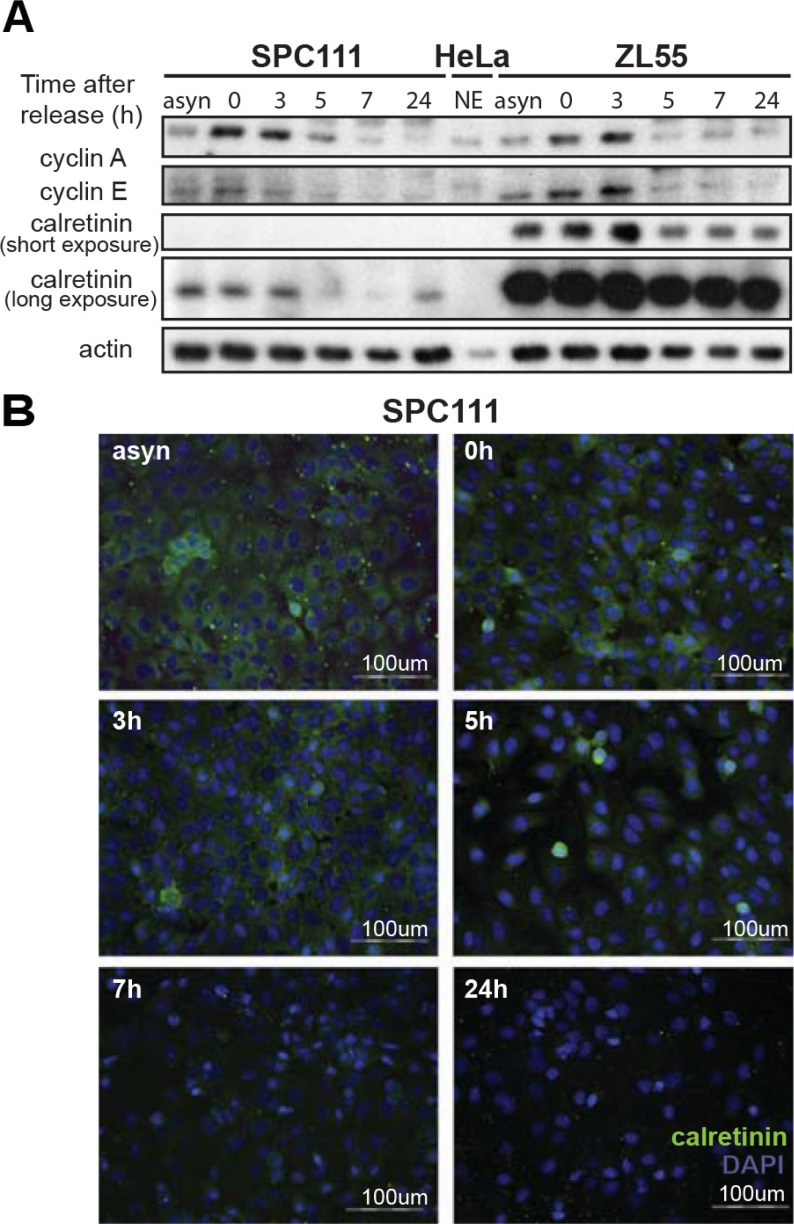
Cell cycle-dependent regulation of calretinin expression (**A**) Calretinin levels in protein lysates from ZL55 and SPC111 synchronized cells collected at different time points (0 h, 3 h, 5 h, 7 h, 24 h) after removal of thymidine block. Analysis of cyclin A and cyclin E expression was used as a reference for cell cycle progression. A nuclear extract of HeLa cells is used to identify the position of the bands of cyclin A and cyclin E Due to the considerably different calretinin expression levels of ZL55 and SPC111 cells, two different exposure times are presented. (**B**) Immunofluorescence analysis of SPC111 cells using calretinin antibody (green) at the same time points as in (A) Cells nuclei are stained with DAPI (blue), and calretinin immunofluorescence is shown in green.

Besides NRF-1, members of the E2F family were among the most frequently identified putative transcription factors binding to the −161/*+*80bp *CALB2* promoter region and E2F2 binding was detected by ChIP analysis. Since E2F family members regulate cell cycle-dependent expression of many genes [28], we hypothesized that calretinin expression might be regulated in a cell cycle-dependent manner. To investigate the kinetics of calretinin expression, ZL55 and SPC111 cell were exposed to double thymidine treatment followed by nocodazole treatment, after which calretinin expression was assessed at different time points (Figure 6A and 6B). The progression through the cell cycle was monitored by flow cytometry analysis (data not shown), while the expression pattern of cyclin A and cyclin E (Figure 6A) served to document the transition of cells from G1/S to M phase [29, 30]. Double thymidine treatment synchronized the majority of cells at the G1/S transition, where high levels of calretinin expression were observed (Figure 6A). Calretinin expression then significantly decreased 5 h after thymidine removal when the majority of cells were in the G2/M phase. The same kinetics of transient and cell cycle-dependent calretinin expression was observed by immunofluorescence (Figure 6B), suggesting that calretinin expression is cell cycle- dependent, being upregulated in G1/S phase of the cell cycle.

## DISCUSSION

In the present study we found *cis*-regulatory sequence elements that are essential for calretinin expression in mesothelioma cells, and identified NRF-1/E2F2 as transcription factors binding to this regulatory sequence.

Mechanisms regulating calretinin expression have been investigated in neuronal cells and colon adenocarcinoma cells, whereas in mesothelioma cells they have not been thoroughly explored yet. A 1.5 kb stretch of the mouse *Calb2* promoter (−1485/*+*60bp) was demonstrated to drive reporter expression in cultured neuronal cell derived from primary embryonic mouse brain tissue [12]. Additionally, a bipartite butyrate-responsive element in the human *CALB2* promoter was found to act as a repressor element upon butyrate treatment in colon carcinoma but not in mesothelioma cells [16]. However, specific *cis*-regulatory elements positively regulating calretinin expression had not yet been identified.

Genomic sequence surrounding transcription start site of *CALB2* gene contains a GC-rich region indicating a possible role for DNA methylation in regulating calretinin expression. However, 5-Aza-CdR, a DNA hypomethylating agent, did not increase calretinin expression in low-calretinin expressing SPC111 cells. It even resulted in a decrease in calretinin expression levels indicating that a certain degree of methylation is required for optimal/maximal *CALB2* transcription. Additionally, we did not observe differential methylation status of *CALB2* promoter between epithelioid and biphasic mesothelioma in tumors from TCGA database or a strong negative correlation of promoter methylation with gene expression. Thus altered DNA methylation of the *CALB2* promoter is very likely not responsible for the differences in calretinin expression levels between low-expressing biphasic/sarcomatoid and high-expressing epithelioid mesothelioma cells.

We further characterized a stretch of~1 kb (−835/*+*80bp) of the human *CALB2* promoter by creating four different 5′-deletion promoter reporter constructs and testing their activity in four different mesothelioma cell lines. The −161/*+*80bp fragment of the *CALB2* promoter was identified as the minimal element resulting in sustained transcriptional activity in all tested mesothelioma cell lines. This is consistent with the previously defined minimal mouse *Calb2* promoter (−115/*+*54bp) shown to be active in rat cortical neuronal cells, rat cerebellar granule cells, colon adenocarcinoma (WiDr) and mesothelioma cells (SPC111) [13, 14]. Moreover, we found that the transcriptional activity of the −161/*+*80bp *CALB2* promoter reporter was highly correlated with *CALB2* expression levels. We are aware that using as normalizer pRL-TK plasmid, which carries the Renilla luciferase gene under the HSV-1 TK promoter might theoretically be affected by different levels of Specificity protein 1 (Sp1) [31]. Rao et al. reported recently overexpression of Sp1 in mesothelioma compared to normal mesothelium [32]. However, the variation of Sp1 levels between the lowest and the highest expressing mesothelioma cell line investigated was 4-fold. Since we observed an about 30-fold difference in *CALB2* promoter activity between the different mesothelioma cell lines, we can reasonably assume that the difference is not due to differential expression of the normalizer.

*In silico* analysis of the human −161/*+*80bp *CALB2* minimal promoter region revealed a series of potential functional transcription factors (TF) among which NRF-1, E2F2 and CREB were of greatest interest for the following reasons. NRF-1 is a transcription factor implicated in mitochondrial biogenesis and function [33] and was predicted as TF binding to the *CALB2* promoter [34]. Computer-assisted identification predicted *CALB2* to be an E2F target gene as well [35]. Finally, CREB had been demonstrated as an asbestos activated TF in human mesothelial cells [26]. Mutation of E2F2 (−69), NRF-1 (−41), two NRF-1 (−41;–35) and CREB (−2) predicted binding sites significantly reduced *CALB2* promoter activity in ZL55 and SPC111 cells. Mutations in the E2F2/NRF-1 (−64;-63;-61) putative binding sites (GA**GCGCG*CG*C*G*C**CCCCT; bold indicates NRF-1 consensus, underlined are the nucleotides mutated in the functional assay) led to a 70–80% decrease in the promoter activity revealing this sequence as the most important *cis*-acting site. In the studies of Billing-Marczak et al. [13, 14], an essential element for the promoter activity in the mouse *Calb2* promoter (−115/*+*54bp) was located at the −91/−80bp position and identified as an “AP-2 like” sequence (**CGC**CCCCTTCCG, in bold are nucleotides overlapping with NRF-1 consensus, underlined are the nucleotides that had been mutated in the functional assay). Although *trans*-acting factors were not identified, functional inactivation in neuronal cells was obtained by mutating one nucleotide also present in the E2F2/NRF-1 putative binding site. The same mutation did not abolish promoter activity in cancer cells [14]. This could be interpreted in two ways: either mutating a single base in the E2F2/NRF-1 binding site is not enough to abolish activity in cancer cells or different transcription factors are operational in neuronal and cancer cells.

A nucleotide sequence analysis revealed that the E2F2/NRF-1-site in the *CALB2* promoter region displays a high degree of identity across different species. An EMSA assay revealed three different DNA-protein complexes and chromatin immunoprecipitation assay showed the presence of both NRF-1 and E2F2 bound to *CALB2* promoter. This is consistent with a previous study demonstrating that NRF-1 is a co-regulator of E2F family members target genes [27]. For all these reasons and since we observed similar expression of NRF-1 in a panel of mesothelioma cell lines with different calretinin expression levels, it is most likely that NRF-1 is a part of a protein complex that altogether activates calretinin expression. Identified proteins interacting with NRF-1 include SP4 (specificity protein 4) [36]. Interestingly, the *in silico* analysis on −160/*+*80bp *CALB2* promoter region predicted a SP4 binding site located next to the E2F2/NRF-1 site. SP4 expression is tissue specific, primarily expressed in the brain, some epithelial tissues, testis and developing teeth [37] and its coordinate activity with NRF-1 has been described to couple energy generation and neuronal activity [38]. Further studies should investigate whether SP4 is involved in the regulation of calretinin expression.

E2F factors are known to regulate S-phase entry [39]. Consistent with E2F regulating *CALB2* promoter activity, calretinin expression was found to be cell cycle-dependent with a maximum level of expression at G1, and progressively decreased during cell cycle progression. Since calretinin expression is associated with better clinical outcome [7–9], it would be interesting to investigate whether tumor cells positive for calretinin are growth arrested cells.

Knowledge about mechanisms regulating NRF-1 and E2F would help for a better understanding of the complex biology of mesothelioma, where loss of calretinin expression during epithelioid tumor progression is associated with worst outcome (Vrugt et al., ms in preparation).

Investigation of the promoter of another MPM marker, mesothelin, has led to the discovery of a cancer-specific element driving mesothelin overexpression in cancers [40]. In the transgenic MexTAg mouse model, where the mesothelin promoter is placed upstream of the large T antigen, the promoter becomes active in mesothelial cells after asbestos exposure, leading to asbestos induced mesothelioma [41]. This suggests that different transcription factors act on the mesothelin promoter in normal cells and cancer cells. In several studies aiming at driving suicide gene expression in cancer cells, a 2.2kb human *CALB2* promoter fragment was reported not to be specific for cancer cells [42, 43]. In our study, we describe a *CALB2* promoter fragment (−161/*+*80bp) with activity proportional to calretinin expression and identified NRF-1/E2F2 as specific *trans*-activating factors. Future studies will assess whether this promoter activity is differently regulated in cancer and neuronal cells.

Although this work cannot directly exclude a role of post-transcriptional mechanisms such as RNA stability in regulating calretinin expression in mesothelioma cells, this is the first study defining *trans*-activating factors in a gene that is not only a diagnostic and prognostic marker, but also has a functional role in MPM. Our experiments demonstrate that calretinin expression is activated at the transcriptional level by NRF-1 and E2F2 transcription factors and that expression levels are cell cycle-dependent. Given recent findings about E2Fs involvement in processes such as regulation of cell fate, occasionally linked to metabolic adaptation [44], our results on the regulation of calretinin expression are expected to have a persistent impact for the understanding of MPM development.

## MATERIALS AND METHODS

### Cell culture

The following mesothelioma cell lines have been used: ZL55, ZL5, ZL34 and SPC111 from our laboratory [45]; NCI-H226, MSTO-211H, MeT5A [46] were obtained from ATCC; ACC-MESO-4 [47] were obtained from Riken BRC; and MERO-82, MERO-83, MERO-84 [48] and ONE58 [49] were obtained from the European Collection of Cell Cultures. MPM cells established in our laboratory were maintained as described by Thurneysen et al. [50] and were authenticated by DNA fingerprinting of short tandem repeat loci (Microsynth, Switzerland). The other cell lines were cultured in D-MEM-F12 supplemented with 15% FCS and 1% Penicillin/Streptomycin solution. HEK293 cells were cultured in DMEM high glucose medium supplemented with 10% FCS and 1% penicillin/streptomycin. All cells were cultured at 37°C in a humidified 5% CO_2_ atmosphere.

### Gene expression analysis

RNA extraction using Qiagen RNAeasy^®^ and cDNAs was prepared from 400–500 ng of total RNA (Qiagen QuantiTect^®^ Reverse Transcription protocol) [51]. Selected gene expression analysis using MIQE [52] compliant protocols was conducted as previously described [51]. Briefly, cDNA was amplified by the SYBR-Green PCR assay and products were detected on a 7900HT Fast real-Time PCR system (SDS, ABI/Perkin Elmer). Relative mRNA levels were determined by comparing the PCR cycle thresholds between cDNA of a specific gene and histone (ΔCt method). The following primers were used: calretinin (5′-GCGAACCGGCCGTACGATGA-3′ and 5′-AGAGGCCCAATTTGCCATCCCCG-3), Mage-C1 (5′-GGGATGTGCTGAGTGGAATAG-3′ and 5′-CTCCC GGTACTCTAGGTAATGT-3′), CTAG1B (NY-ESO-1) (5′-GTCCGGCAACATACTGACTATC-3′ and 5′-GTGA TCCACATCAACAGGGAA-3′).

### Western blot analysis

Total protein extracts were prepared by lysing the cells with hot Laemmli sample buffer (60 mM Tris-Cl pH 6.8, 100 mM DTT, 5% glycerol, 1, 7% SDS) and pressed few times through syringes (26 G). Protein concentration was determined using a Pierce^™^ 660nm Protein Assay (Thermo Scientific). A total of 5 μg protein per extract was separated on denaturing 10–20% gradient SDS-PAGE gels. Proteins were transferred on PVDF transfer membranes (0.45 μm, Perkin Elmer). For Western blotting, membranes were probed with the following primary antibodies: anti-calretinin (Sigma, HPA007306), anti-cyclin E (Santa Cruz Sc-247), anti-cyclin A (Millipore, 06–138), anti-NRF-1 (Santa Cruz SC33771, H300) and mouse anti-actin (#69100) from MP Biomedicals. Membranes were then incubated with the secondary antibody rabbit anti-mouse IgG-HRP (A-5420) from Antell, and goat anti-rabbit IgG-HRP (#7074) from Cell Signaling. The signals were detected by enhanced chemiluminescence (ECL^™^ Western Blotting Reagents, GE Healthcare) and detected on photosensitive film (Super RX Fuji x-Ray Film, Fujifilm). Relative quantification was assessed using ImageJ (NIH).

### 5-aza-2′deoxycytidine treatment of cells

On the first day, 2.5 × 10^4^ cells were seeded in a 6-well plate and on the next day the medium was replaced with the medium containing 100 nM and 250 nM of 5-Aza-2-CdR. After 24 h, the drug containing medium was replaced with the fresh medium. On the fifth day, cells were treated again with 5-Aza-2CdR for 24 h and then the medium was replaced by fresh one. Cells were kept in culture for another 24 h after which samples were collected for RT-PCR and Western blot analysis.

### *In silico* analysis

Analysis of the 241bp of the promoter sequence for potential functional transcription factors was carried out using MatInspector software (Genomatix Software GmbH, Munich, Germany) [25].

Level 3 normalized DNA methylation data and clinical information was downloaded from The Cancer Genome Atlas (TCGA) data portal on August 10th, 2015. Normalized *CALB2* gene expression values (RNAseq) from the same TCGA samples were downloaded from the UCSC cancer genome browser on August 10th, 2015. There were 87 mesotheliomas with available Illumina HumanMethylation450 methylation, gene expression, and clinical data. The majority of tumors were epithelioid histology (*n =* 57), with *n =* 23 biphasic, *n =* 5 diffuse not otherwise specified and *n =* 2 sarcomatoid tumors. Patient demographic and tumor characteristics are provided in Supplementary Table 1.

### Plasmids constructs and side-directed mutagenesis

To investigate the control of *CALB2* transcription, 838bp, 419bp, 216bp and 161bp upstream and 80bp downstream of the *CALB2* transcription starting site were amplified from genomic DNA ZL55 cells using following primers: F: 5′-ACGT ^NheI^GCTAGC ^−838^ACATTCCCACGATGTCCCTA-3′ (Addgene, 66750); F: 5′- ACGT^NheI^GCTAGC^−419^AGCTCTTCCTGCTGTGGA AGGATCAGAA-3′ (Addgene, 66747); F: 5′-ACGT^NheI^GC TAGC ^−264^AAGTGGTGGATGTACTCAAG-3′ (Addgene, 66746); F:5′- ACGT ^HindIII^AAGCTT ^−161^TCTCAGCGCA GAGGTAAGGG-3′ (Addgene 66745)- along with the reverse primer: R: 5′-ACGT ^HindIII^AAGCTT ^+155^CC ATATTTCCAGGAACTGGGACGCCGTC-3′. In order to avoid ORF shifts due to the start codon of *CALB2* gene but still to include the 5′-UTR, NcoI restriction sites in 3′ site of the PCR product along with the restriction site within the forward primer (5′) were used to subclone amplicons into the pGL3-basic vector. To obtain constructs with specific mutations, the QuikChange II Site-Directed Mutagenesis kit (Stratagene) was used (mtE2F (−69)-, Addgene 66743; mtCREB (−2), Addgene 66751; mtNRF-1 (−41), Addgene 66744, dmtNRF-1 (−41;–35)-, Addgene 66741, E2F2/NRF-1 (−64), Addgene 66742).

### Transient transfections and reporter assays

To investigate *CALB2* transcription, the dual luciferase assay was used. Briefly, the different constructs were transfected together with Renilla Luciferase (50:1) in cells seeded in 12 wells (100'000 cells/well) as previously described [53]. After 48 h, cells were lysed and analyzed using Dual-Luciferase reporter assay system according to manufacturer's instruction (Promega, Madison, Wi, USA).

### Electrophoretic mobility shift assay (EMSA)

Nuclear extracts were prepared using the NE-PER Nuclear and Cytoplasmic Extraction Reagent (Pierce) following the manufacturer's protocol. The binding reaction was performed by incubating 5 μg of nuclear extract with 125fmol biotin labelled oligonucleotide (5′-CCCTGAGCGCGCGCGCCCCCTTCTG-3′) in the presence of 10 mM Tis-HCl pH8.0, 25 mM NaCl, 10 mM DTT, 5% glycerol, 10 mM MgCl, 2 μM ZnCl, 1 μg salmon sperm dsDNA, 1%NP-40 and incubated on ice for 50 min. For competition reactions, the nuclear extracts were incubated with the specific unlabelled oligonucleotides for 10 min prior to addition of biotin-labelled oligonucleotides and incubated 40 min on ice. For the supershift experiments, specific antibodies (NRF-1 ab34682 and E2F2 sc699 ×) were added after 30 min incubation of the nuclear extracts and biotin-labeled probes and incubated another 20 min on ice. The samples were separated on a 6% 0.5 × TBE gel, transferred on a nylon membrane and visualized using the Chemiluminescent Nucleic Acid Detection Module (89880) according to the manufacturer's protocol.

### Chromatin immunoprecipitation (ChIP)

ChIP was carried out according to the protocol previously described [54]. In brief, cultured cells (4 × 10^6^) were treated with 1% formaldehyde to crosslink proteins to DNA. Chromatin was sonicated using a Bioruptor ultrasonic cell disruptor (Diagenode) to shear genomic DNA to fragments between 100bp and 200bp. Chromatin (20 μg) was diluted tenfold with immunoprecipitation buffer (16.7 mM Tris-HCl (pH 8.1), 167 mM NaCl, 1.2 mM EDTA, 0.01% SDS and 1.1% Triton X-100) and then immunoprecipitated overnight with the corresponding antibodies (NRF-1 Abcam ab34682 and E2F2 Santa Cruz sc699X). After washing, elution and de-crosslinking, the precipitated DNA was purified with phenol-chloroform, ethanol precipitated and quantified by quantitative PCR. Following primers were used: *CDC25A* (5′-TTTCGCGGTAATAGCGGCTC-3′ and 5′-TAGCTGCCATTCGGTTGAGAG-3′) and *CALB2* (5′-AGGTAAGGGCCCTCTAGGAGT-3′ and 5′-CTGCCC TTATATACGCTCTGGA-3′).

### RNA interference by siRNA

For the down-regulation of NRF-1 with small interfering RNAs (siRNA), SPC111 cells were transfected with 25 nM Qiagen smartpool or individual siRNAs targeting NRF-1 or control non targeting (NT) siRNA (Thermo Scientific Dharmacon), according to the manufacturer's reverse transfection protocol as previously described [53]. 3 × 10^4^ cells were then plated in and whole cell lysate was prepared after 72 h.

### Cell synchronization

Cells were synchronized at the G1/S and G2/M border using a thymidine double-block/nocodazole release protocol [55]. Briefly, cells were seeded in 6-well (5 × 10^4^ cells/well) then 24 h later were treated with 2 mM thymidine for 12 h and released for 12 h in fresh medium before the second block was performed for another 12 h with 2 mM thymidine. Cells were then washed three times in phosphate buffer saline (PBS) and either collected (hours after treatment *=* 0) or released in fresh medium containing 40 ng/ml nocodazole to induce mitotic arrest (G2/M) for different time periods.

### Immunofluorescence

SPC111 cells were grown on 12-mm glass coverslips in 24-well plates. Using the same synchronization protocol described above, asynchronized and synchronized cells were fixed for 10 min with 4% paraformaldehyde and permeabilized with 0.05% saponin for 5 min. The cells were then incubated over night at 4 C with an anti-calretinin antibody diluted in PBS containing 1% bovine serum albumin. Secondary antibodies, Alexa Fluor 488- conjugated goat anti-rabbit IgG (Life Technologies) antibody was added for 1 h at RT. Nuclear DNA was stained using DAPI. Coverslips were mounted using Prolong Gold antifade reagent (Life Technologies). Images were acquired using an Olympus B × 61 microscope (Schwerzenbach, Switzerland) equipped with an F-view camera for conventional fluorescence imaging. The image capture was controlled with the AnalySISPro software (Soft Imaging System, Münster, Germany).

### Statistics

Data are expressed as mean ± standard deviation of multiple experiments. Statistical analysis was performed using Mann-Whitney tests using StatView 5.0.1 (SAS institute). Spearman's correlation tests were used to compare TCGA promoter methylation versus gene expression levels. Differences were considered statistically significant at *p* < 0.05.

## SUPPLEMENTARY MATERIALS FIGURES AND TABLES


