# Supplementary material for: Identification of cis- and trans-acting elements regulating calretinin expression in mesothelioma cells
Source: Oncotarget. 2016 Feb 1;7(16):21272–86. doi: 10.18632/oncotarget.7114 (PMC5008284; doi:10.18632/oncotarget.7114)
Supplement: Supplementary file 1 [file oncotarget-07-21272-s001.pdf]

Identification of *cis*- and *trans*-acting elements regulating calretinin expression in mesothelioma cells

Supplementary Materials

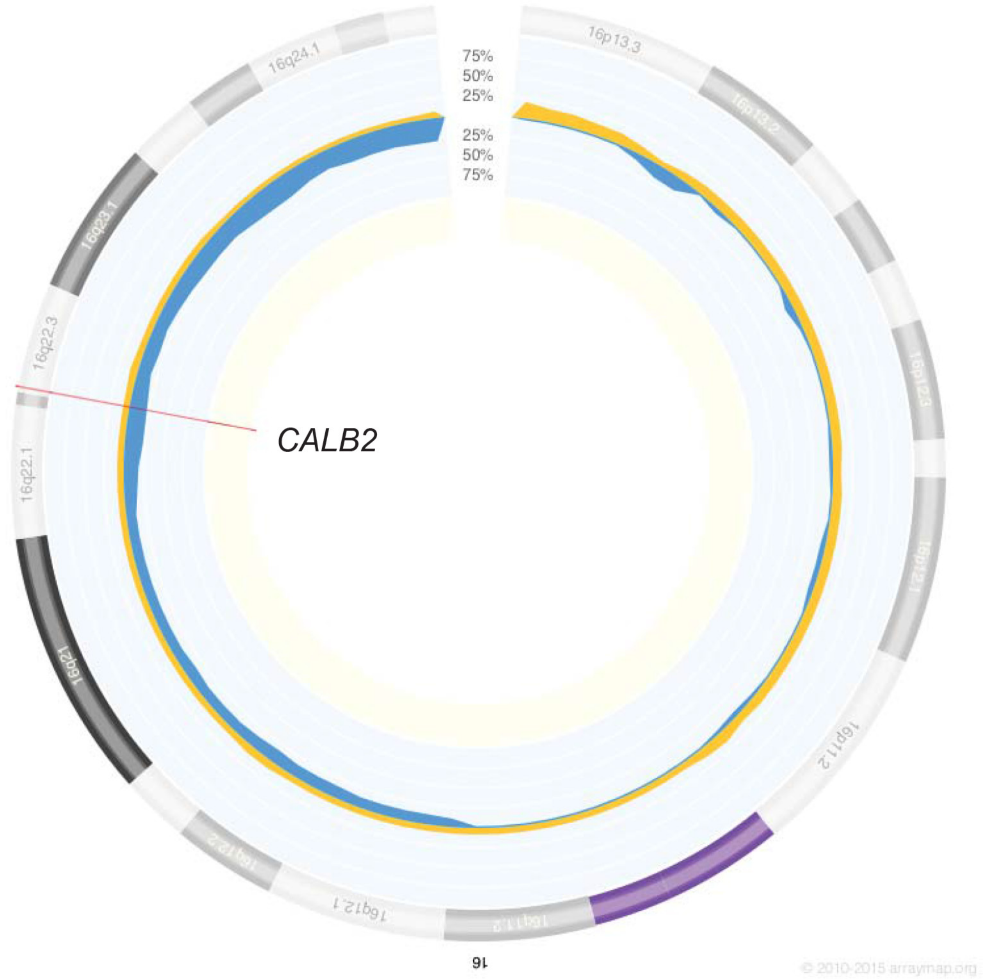

Supplementary Figure S1: Genetic rearrangement profile of chromosome 16 analyzed on mesothelioma samples using arrayMap, a curated reference database and bioinformatics resource targeting copy number profiling data in human cancer, including mesothelioma. The plot represents 49 mesothelioma tumor samples (1, 2), of which 60% are epithelioid, 20% biphasic and 20% sarcomatoid. Percentage values represent frequency of the copy number gains (yellow) and losses (blue) account for the mesothelioma whole data set. Locus of *CALB2* gene is affected by copy number loss in less than 25% of analyzed mesothelioma samples.

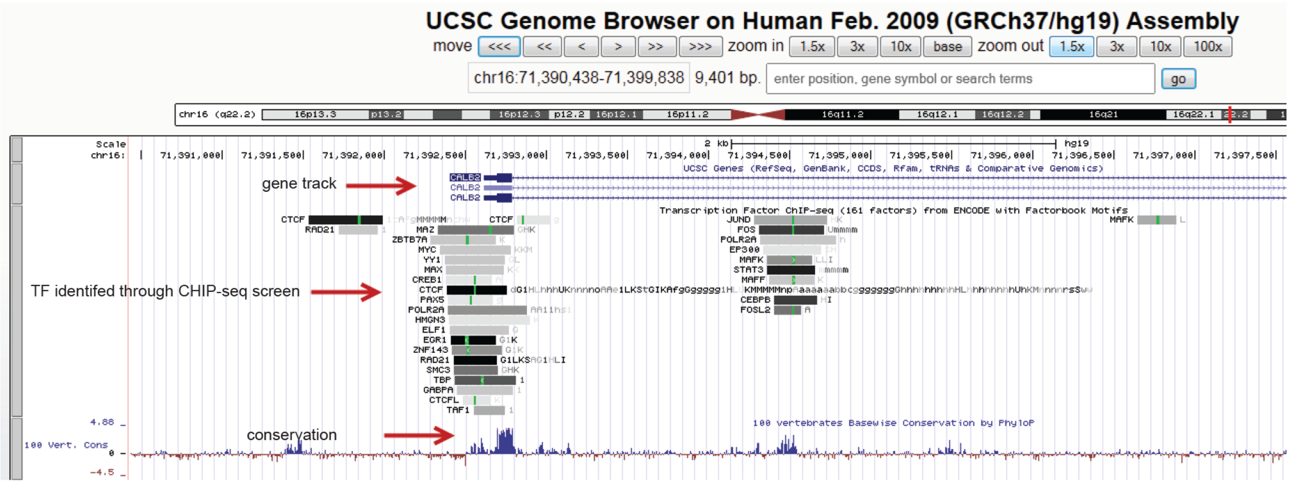

Supplementary Figure S2: ~1 kb of *CALB2* promoter occupied by various transcription factors identified through ChIP-seq screen. UCSC genome browser shows the presence of general TF, Polymerase II, suggesting location of *CALB2* promoter.

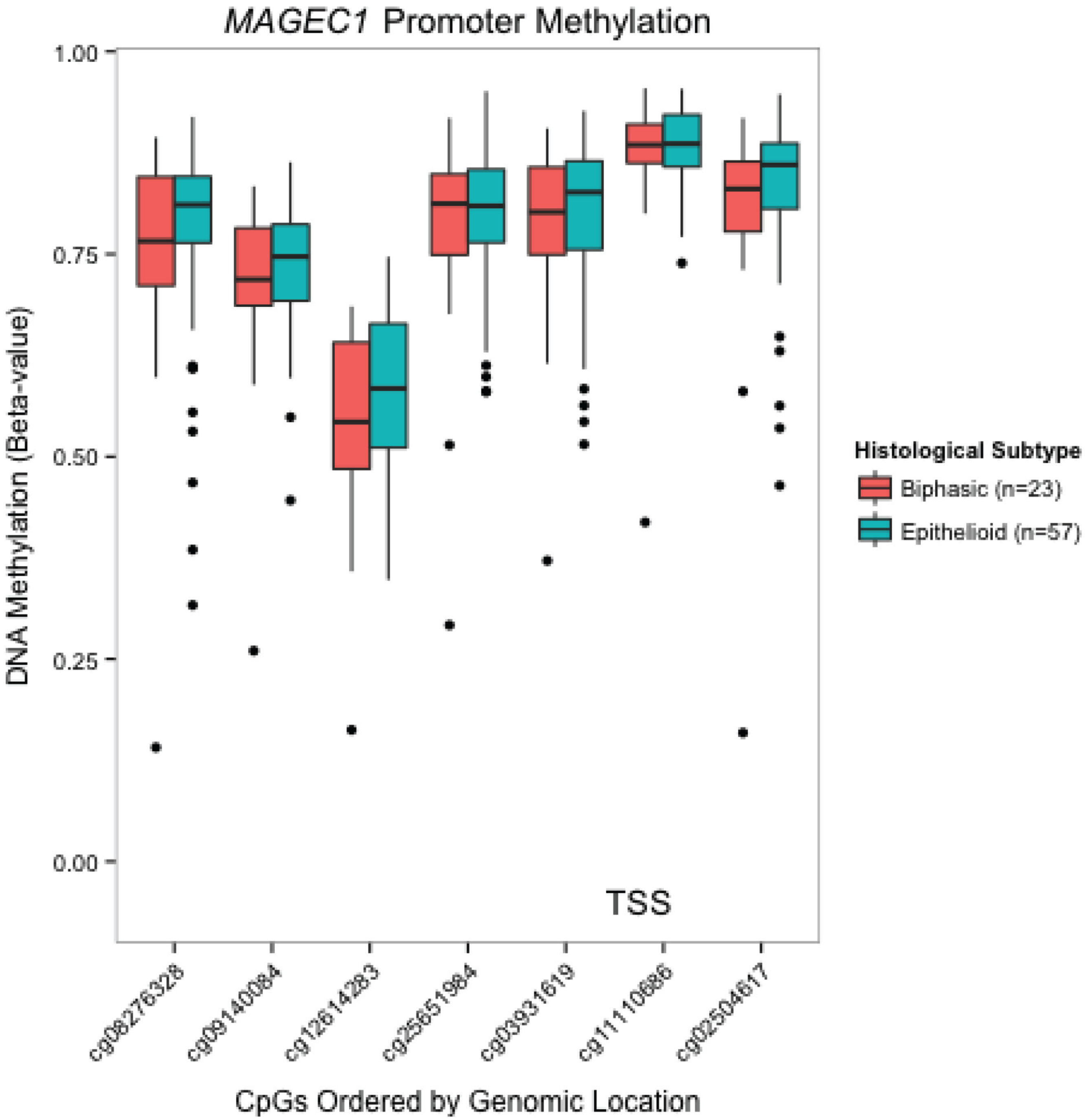

Supplementary Figure S3: *MAGEC1* gene promoter is methylated in both epithelioid and biphasic mesothelioma. CpG sites in the promoter region of *MAGEC1* from the Illumina HumanMethylation450 array are shown in 5′ to 3′ order versus methylation beta-value which represents the percent methylation of the sample. Mesotheliomas are stratified by tumor histology, epithelioid (n = 57), and biphasic (n = 23).

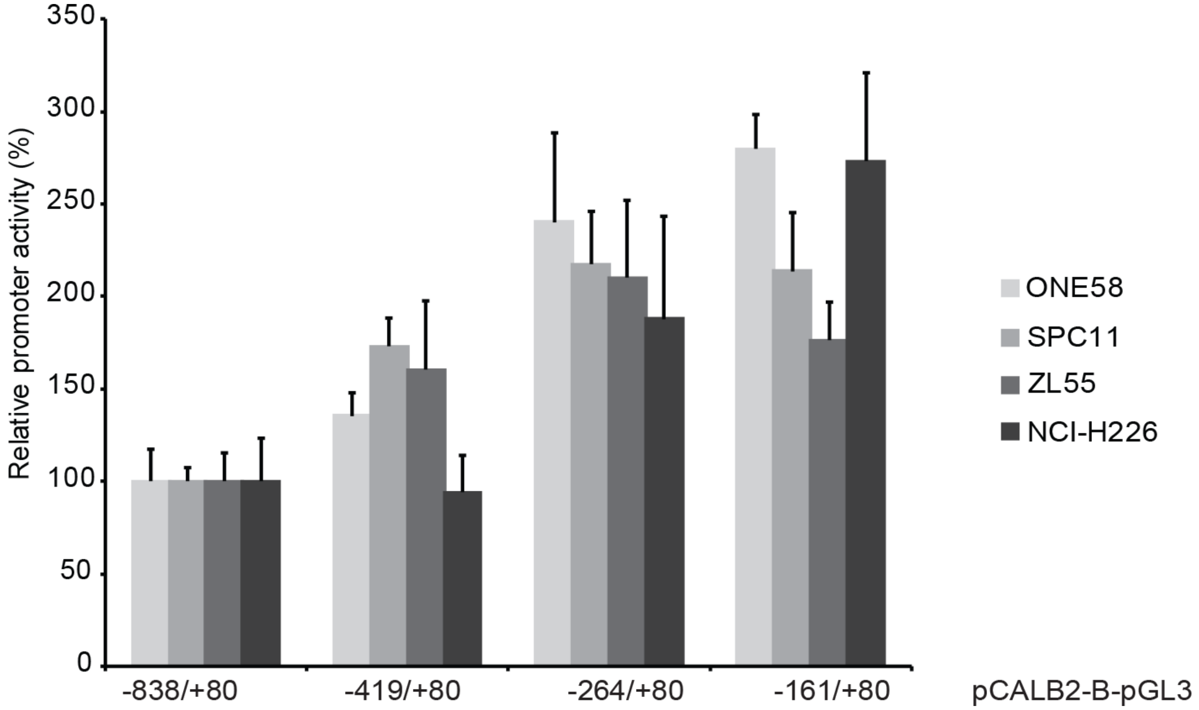

Supplementary Figure S4: The –161/+80bp *CALB2* promoter region has the highest transcriptional activity. Transcriptional activity of the indicated promoter constructs expressed relative to the longest *CALB2* promoter construct, –838/+ 80bp. The –161/+ 80bp region is the minimal promoter.

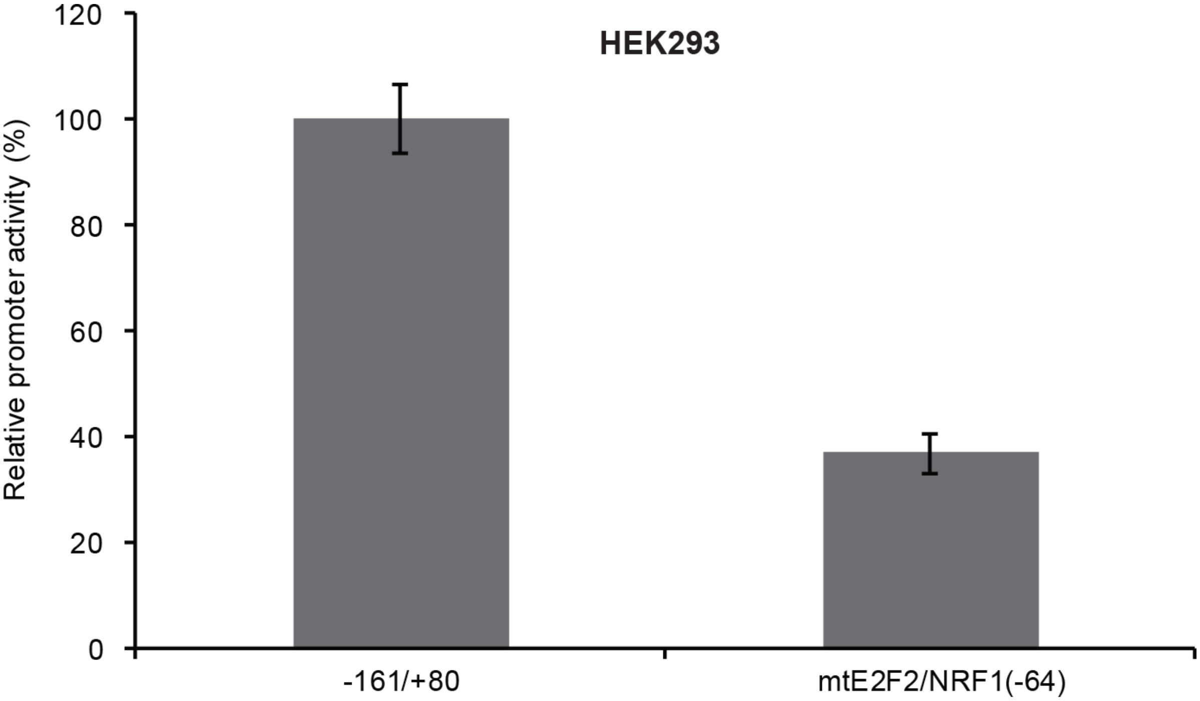

Supplementary Figure S5: The –161/+80bp *CALB2* minimal promoter region is active in HEK293 cells and mutation in the E2F2/NRF-1 site strongly reduced its activity. The wild-type reporter plasmid expression was arbitrarily set to 100% and the reporter activity of the mutants is expressed as a percentage of the wild-type construct. Mea*n* ± SD, *n* = 3.

```
human           TCTCAGCGCAGAGGT-AAGG-GCCCTCTAGGAGTCCGGGCCGAGCCTCTCGCGCCGCCGC
chimpanzee      TCTCAGCGCAGAGGT-AAGG-GCCCTCTAGGAGTCCGGGCCGAGCCTCTCGCGCCGCCGC
orangutan       TCTCAGCGCAGAGGT-AAGG-GCCCCCTAGGAGTCCGGGCCGAGCCTCTAGCGCCGCCGC
baboon          -CTCAGCGCAGAGGTAAGGG-CCCCCCTACGAGTCCGGGCCCAGCCTCTCGTGCCGCCGC
rat             TCTCATTGAAGACACTCTCCTGGTCCCCAGGAACCCTGGCAG----AGAGGAGCCTCCGC
mouse           --------------CTCTCCCGGTCCCCAGGAACCCTGGCAG----AGAAGAGCCCCCGC
                                        * * * **  ** ***          * *** ****
                                                   E2F2/NRF-1
human           CCCCGCCGCGCCGCGCCCCGGTCGGATTCCCTGAGCGCGCGCGCCCCCTTCTGGCGGCCG
chimpanzee      CCCCGCCGCGCCGCGCCCCGGTCGGATTCCCTGAGCGCGCGCGCCCCCTGCTGGCGGCCG
orangutan       CCCCGCCGCGCCGCGCCCCGGTCGGATTCCCTGAGCGCGCGCGCCCCCTGCTGGGGGCCG
baboon          CCTCGCCGCGCCGCGCCCCGGCCGGATTCCCTGAGCGAGCGCGCCCCCTGCTGGCAGCCG
rat             CCGTGCAGCGCC-GCGCCCTGCGCGATTCCCTGAGTGTGCGCGCCTCCTTCCGGCGGCCC
mouse           CCGTGCAGCGCC-GCGCCCTGCGCGATTCCCTGAGTGTGCGCGCCCCCTTCCGGCGGCCC
                **  ** *****    *** *   *********** * ******* *** * **  ***

human           GGCGCAGGCGCAGGCTCCAGAGCGTATATAAGGGCAGCGTGGCGCACAACCCCAGCGCGA
chimpanzee      GGCGCAGGCGCAGGCTCCAGAGCGTATATAAGGGCAGCGTGGCGCACAGCCCCAGCGCGA
orangutan       GGCGCGGGCGCAGGCTCCAGAGCGTATATAAGGGCAGCGTGGCGCACAGCCCCAGCGCGA
baboon          GGCGCGGGCGCAGGCTCCAGAGCGTATATAAGGGCAGCGTGGCGCACAGCCCCAGCGCGA
rat             AACGC--GGTGCAGCTCCGGGCTGCATATAAAGGCAGCGTGGCGCGCAGCTCCAGCGCGA
mouse           GACGC--GGCGCAGCTCCGGGCTGCATATAAAGGCAGCGTGGCGCGCAGCCCCAGCGCGA
                  *** *     ***** *   * ****** ************* ** * *********

human           GTGCCAGAGCCCAGCCGGCGCGGAGCGGGAGCGGTGCAGGCTGAGGTCTCCGA-------
chimpanzee      GTGCCAGAGCCCAGCCGGCGCGGAGCGGGAGCGGTGCAGGCTGAGGTCTCCGA-------
orangutan       GTGCCAGAGCCCAGACGGCGCGGAGCGGGAGCGGTGCAGGCTGAGGTCTCCGA-------
baboon          GTGCCAGAGCCCAGCCGGCGCGGAGCGGGAGCGGTGCAGGCTAAGGTCTCCGA-------
rat             GAACCAGAGCCAAGCGGCACCGAGTGACAGCGCTGAGAGGC------TTAAGATCTCCGG
mouse           GAACCAGAGCCAAGCGGCACCGAGTGACAGCGCGCTGAGAGAGAGGCTTAAGATCTCCGG
                *  ******** **  *   **               **         *  **

human           -GCGGCTCGCC
chimpanzee      -GCGGCTCGCC
orangutan       -GCGGCTCGCC
baboon          -GCGGCTCGCC
rat             AGCCGCTCGCC
mouse           AGCGGCTCGCC
                 ** *******
```

Supplementary Figure S6: NRF-1/E2F2 binding region is highly conserved within *CALB2* promoter. Nucleotide sequence comparison reveals regions with high homology in the *CALB2* promoter of different species. The analysis was performed using the ClustalW2 software. Asterisks indicate conserved nucleotides.

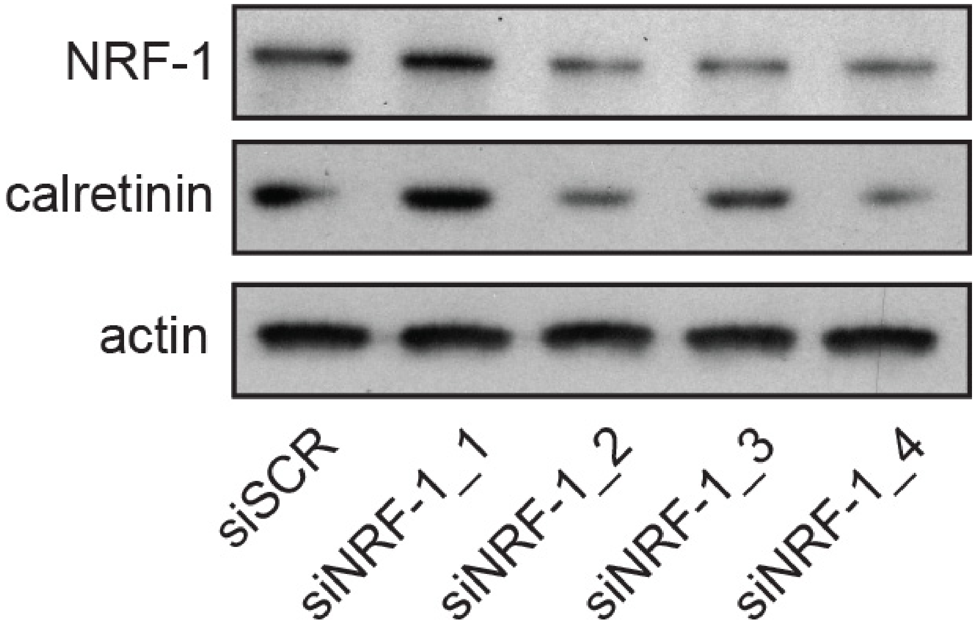

Supplementary Figure S7: Silencing of NRF-1 with siRNA decreases calretinin expression in SPC111 mesothelioma cells.

Supplementary Table S1: Patient demographic and tumor characteristics for methylation analysis of *CALB2* promoter

Covariate	*n* (%)
Age at diagnosis	
Range (median)	28–81 (64)
Mean (sd)	63 (9.8)
Gender	
Male	71 (81.6)
Female	16 (18.4)
Race	
Caucasian	85 (97.7)
Asian	1 (1.15)
African American	1 (1.15)
Histology*	
Epithelioid	57 (65.5)
Biphasic	23 (26.5)
Sarcomatoid	2 (2.3)
Diffuse NOS	5 (5.7)
Stage	
I / II	26 (29.9)
III / IV	61 (70.1)

*Epithelioid and biphasic samples were included in data analysis, NOS: not otherwise specified.

Supplementary Table 2: Correlation between *MAGEC1* gene promoter CpG methylation and *MAGEC1* expression (*Spearman's test)

cgID	Chromosome	Genomic Location	Epithelioid Correlation Coefficient	Epithelioid *P*-value*	Biphasic Correlation Coefficient	Biphasic *P*-value*
cg08276328	X	140990738	–0.26	0.05	0.09	0.67
cg09140084	X	140990878	–0.11	0.4	0.13	0.55
cg12614283	X	140991045	–0.14	0.28	0.09	0.68
cg25651984	X	140991592	–0.19	0.15	0.28	0.2
cg03931619	X	140991599	–0.07	0.6	0.27	0.21
cg11110686	X	140991714	–0.29	0.03	0	0.99
cg02504617	X	140992417	–0.29	0.03	0.15	0.5

REFERENCES

1. Lindholm PM, Salmenkivi K, Vauhkonen H, Nicholson AG, Anttila S, Kinnula VL, et al. Gene copy number analysis in malignant pleural mesothelioma using oligonucleotide array CGH. Cytogenet Genome Res. 2007; 119:46–52.
2. Christensen BC, Houseman EA, Poage GM, Godleski JJ, Bueno R, Sugarbaker DJ, et al. Integrated profiling reveals a global correlation between epigenetic and genetic alterations in mesothelioma. Cancer Res. 2010; 70:5686–94.
